# What RNAi screens in model organisms revealed about microbicidal response in mammals?

**DOI:** 10.3389/fcimb.2014.00184

**Published:** 2015-01-12

**Authors:** Prasad Abnave, Filippo Conti, Cedric Torre, Eric Ghigo

**Affiliations:** CNRS UMR 7278, URMITE, IRD198, INSERM U1095, Aix-Marseille UniversitéMarseille, France

**Keywords:** *D. melanogaster*, *C. elegans*, *D. rerio*, RNA interference, host-pathogen interaction, innate immunity, orthologs

## Abstract

The strategies evolved by pathogens to infect hosts and the mechanisms used by the host to eliminate intruders are highly complex. Because several biological pathways and processes are conserved across model organisms, these organisms have been used for many years to elucidate and understand the mechanisms of the host-pathogen relationship and particularly to unravel the molecular processes enacted by the host to kill pathogens. The emergence of RNA interference (RNAi) and the ability to apply it toward studies in model organisms have allowed a breakthrough in the elucidation of host-pathogen interactions. The aim of this mini-review is to highlight and describe recent breakthroughs in the field of host-pathogen interactions using RNAi screens of model organisms. We will focus specifically on the model organisms *Drosophila melanogaster, Caenorhabditis elegans*, and *Danio rerio*. Moreover, a recent study examining the immune system of planarian will be discussed.

## Introduction

The innate immune system is the first line of defense against invading pathogens and is therefore considered to be of prime importance in host-pathogen interaction studies. Various model organisms have been studied for many years to understand the role of various components of the innate immune system pathways involved in host-pathogen interactions. Several strategies are available to identify the role of genes involved in various pathways. Gene knock-outs or mutations have been used routinely to identify the functions of genes. However, these strategies are limited by several constraints; for example, the number of genes that can be targeted, two or three at a time, is highly limited. The development of RNA interference, a post-transcriptional gene silencing technique in small model organisms, represents a major breakthrough in these types of studies. When the RNAi system was discovered in *Caenorhabditis elegans* by Fire and Mello in 1998, the scientific community soon realized the potential of this tool for research (Fire et al., 1998). Later, when it was confirmed that RNAi also operates efficiently in *Drosophila* (Hammond et al., 2000), large-scale gene silencing strategies were developed for *Drosophila* cells (Ramet et al., 2002; Kiger et al., 2003; Lum et al., 2003; Boutros et al., 2004). The RNAi technique is associated with several limitations and shortcomings such as, transient and incomplete inhibition and even if there is sufficient down regulation of gene the phenotype can differ from the genetic null phenotype. It has also been observed that RNAi may not elicit effective and specific inhibition in all situations and may have nonspecific and off-target effects. However, despite some important limitations such as the lack of cell-type specifity, RNAi has become one of the highly popular and favorite techniques of researchers for analyzing gene functions. The high-throughput screens carried out in various model organisms have enabled the discovery of several components involved in host-pathogen interactions and thus helped to identify and/or validate the functions of their orthologs present in the mammalian immune system.

In this mini review, we will highlight various components of innate immune pathways involved in multiple stages of host-pathogen interactions, which were discovered from RNAi screenings in *D. melanogaster, C. elegans, D. rerio* and their orthologs in mammals. Finally, a recent study confirming the existence of a genuine innate immune response in planarian will be discussed briefly.

### Drosophila melanogaster

Several *Drosophila* immune response elicitors have been discovered so far (Table 1). Pattern recognition receptors (PRRs) are molecules involved in recognizing microbe specific patterns. They are proteins expressed by cells of the innate immune system (which includes macrophages and neutrophils in mammals) to identify pathogen-associated molecular patterns (PAMPs) that play a role during pathogen defense or cellular stress. They are also known as primitive PRRs, as they evolved before adaptive immunity (Buchmann, 2014). Usually, these molecules induce an antimicrobial signaling cascade in response to microorganisms. Peptidoglycan recognition molecules (PGRPs), which include CD14 (cluster of differentiation 14), TLR2 (Toll-like receptor 2), NOD1, and NOD2 (nucleotide-binding oligomerization domain 1 and 2), and peptidoglycan-lytic enzymes (lysozyme and amidases), are PRRs that are highly conserved in higher eukaryotes, from insects to mammals (Dziarski, 2003).

**Table 1:** **Discoveries in *D. melanogaster, C. elegans* and *D. rerio* using RNAi and the homology of the identified genes in mammals**.

	**Mammalian Orthologs**	***D. melanogaster***	***C. elegans***	***D. rerio***	**References**
Recognition	Glycopeptide hormone receptors		FSHR1		Cho et al., 2007
	PGRPs	PGRP-LC		PGRP-SC1a	Choe et al., 2002; Gottar et al., 2002; Ramet et al., 2002; Li et al., 2007
				PGRP-SC2	
	TOL receptors		TOL-1		Aballay et al., 2003
			Bus-2/2/12/8		Gravato-Nobre et al., 2011
	C-type lectins		C-type lectins		
Signaling	IAPs	Iap2			Foley and O'Farrell, 2004; Gesellchen et al., 2005
		Dnr1			
	PDGF/VEGF receptor	PVR			Ragab et al., 2011
	Myd88			Myd88 (TLR adaptor)	van der Sar et al., 2006
		Deaf-1			Kuttenkeuler et al., 2010
	CNOT4	Not4			Grönholm et al., 2012
	GRK5	Gprk2			Valanne et al., 2010
	Akirin1/2	Akirin			
	Insulin receptor		DAF-2 (Membrane receptor)		Murphy et al., 2003
	FOXO		DAF-16 (Transcription Factor)		Murphy et al., 2003
	P38 MAPK JNK		P38 MAPK JNK		Pukkila-Worley et al., 2012
	TGF-β		TGF-β		Irazoqui et al., 2010
Antimicrobial response	Transglutaminases	Transglutaminases			Shibata et al., 2013
	NADPH oxidase (NOX)			Duox	Flores et al., 2010
	SPP proteins		SPP proteins		Roeder et al., 2010
Cell migration	CXCR3/CXCR5			CXCR3.2 (Chemokine receptor)	Meijer and Spaink, 2011
	MMP9 (Metalloproteinase)			MMP9	Meijer and Spaink, 2011

In *Drosophila*, PGRP-LC, a transmembrane protein required for the response to bacterial infection, was discovered by three separate groups in the year 2002 (Choe et al., 2002; Gottar et al., 2002; Ramet et al., 2002). One of these discoveries was a study by Ramet and colleagues. It involved the first large-scale RNAi screen in *Drosophila* S2 cells, and it demonstrated the potential of the RNAi screening approach. Rosetto and colleagues reported in 1995 that the Toll receptor acts as an immune activator in a *Drosophila* blood cell line, thereby demonstrating the role of the Toll pathway in immunity (Rosetto et al., 1995); moreover, the importance of the Toll receptor for antifungal resistance in *Drosophila* was confirmed the next year (Lemaitre et al., 1996). Unlike in other species, the *Drosophila* Toll receptor acts as a cytokine receptor and not as a PRRs (Rämet, 2012). Kuttenkeuler et al. (2010) performed a genome-wide RNAi screen to identify the transcriptional factors involved in the Toll-dependent immune response and demonstrated that Deformed Epidermal Autoregulatory Factor 1 (DEAF1) is required for the expression of the Toll target gene Drosomycin, both in cultured cells and *in vivo*. They also showed that DEAF1 is required to survive fungal, but not *E. coli*, infection (Kuttenkeuler et al., 2010). Another targeted screen by Huang and colleagues revealed that the endosomal proteins Myopic (MOP) and Hepatocyte growth factor-regulated tyrosine kinase substrate (HRS) are required to activate the Toll signaling pathway both in cultured cells and in flies (Huang et al., 2010). During the same year, Valanne et al. performed genome-wide RNAi screening to find components of the NF-κB (nuclear factor κ-light-chain-enhancer of activated B cells) pathway and identified an evolutionarily conserved G-protein coupled receptor kinase 2 (GPRK2) that interacts with Cactus and regulates the Toll pathway.

The JAK/STAT (Janus kinase and Signal Transducer and Activator of Transcription) pathway is known to play an important role in the control of a wide variety of biological processes. To investigate its mechanisms in the context of infection, two groups (Baeg et al., 2005; Muller et al., 2005) carried out a genome-wide RNAi screen in cultured *Drosophila* cells. Comparison between the screens by Baeg and Muller reveals that although both groups used essentially the same library of dsRNA molecules, they performed the studies under distinct conditions and used different cell lines and reporters, causing very little overlap (5-6%) in their discoveries. During the Muller screen, 73% of the genes identified are positive regulators of the pathway (regulators that enhance the Drosophila JAK/STAT activity), whereas 75% of the genes identified during the Baeg screen are putative negative regulators (regulators that negatively regulate the Drosophila JAK/STAT pathway). Negative regulators were also discovered during *in vivo* genome-wide RNAi screens (Kleino et al., 2005; Cronin et al., 2009) to discover genes implicated in susceptibility or resistance to infection with the bacterium *Serratia marcescens*. The group identified multiple genes involved in antibacterial defense and showed that the JAK-STAT signaling pathway regulates stem cell proliferation, highlighting an essential role of epithelial cell homeostasis in the gut during the immune response (Cronin et al., 2009). Several other screens allowed the identification of essential components of the immune deficiency (IMD) pathway. These screens identified Inhibitor of apoptosis 2 (IAP2) and TAK1-associated binding protein 2 (TAB2/TAB/CG7417) as essential components of the IMD pathway (Gesellchen et al., 2005; Kleino et al., 2005) and demonstrated that the proteolytic activity of DREDD is required to cleave IMD proteins (Paquette et al., 2010). Applying RNAi to target transglutaminases (TGs) in *Drosophila* has demonstrated that TG suppresses the expression of genes encoding IMD-controlled antimicrobial peptides, enabling immune tolerance against commensal microorganisms (Shibata et al., 2013).

Phagocytosis is a specific form of endocytosis involving the vesicular internalization of solids, such as bacteria. Utilizing this phagocytic capability of *Drosophila* S2 cells, several RNAi screenings have been performed to discover various components involved in phagocytosis. Ramet and colleagues identified 34 gene products involved in phagocytosis, including proteins that participate in vesicle transport, actin cytoskeleton regulation and a cell surface receptor, and they demonstrated the involvement of PGRP-LC in phagocytosing Gram-negative bacteria (Ramet et al., 2002). Using *Mycobacterium fortuitum*, factors required for general phagocytosis and infection in *Drosophila* S2 cells have been identified; they are mostly involved in the actin cytoskeleton and vesicle trafficking (Philips et al., 2005). These results have been confirmed by the results of several other studies, identifying host factors required for the pathogenesis of intracellular bacteria, such as *Listeria monocytogenes* (Agaisse et al., 2005), and yeast, such as *Candida albicans* (Stroschein-Stevenson et al., 2006). A comparison of the genes appearing in multiple screens revealed that most of the involved genes encode actin regulatory proteins and vesicle transport proteins, suggesting the importance of these processes for pathogen phagocytosis. Moreover, a recent screen targeting factors required for the phagocytosis of *Leishmania donovani* indicated the importance of the small GTPase RAB5, the RAC1-associated protein SRA1 and the actin cytoskeleton regulatory protein SCAR in parasite phagocytosis (Peltan et al., 2012). Thus different RNAi screenings performed in *Drosophila* as well as in *Drosophila* S2 cells have discovered several components of innate immune system such as pathogen recognition receptors (PGRP-LC), transcriptional factors (DEAF1) involved in immune response, components required for phagocytosis of pathogens, components of IMD and NF-κB pathway, molecules required to activate Toll signaling pathway (MOP, HRS) and also the modulators of JAK/STAT pathway.

### Caenorhabditis elegans

*C. elegans* exhibits a strong host defense response when challenged by different microorganisms (Table 1). For these worms, microorganisms are both a food source and potential pathogens. Although *C. elegans* does not have PGRPs, it nevertheless appears to discern microorganisms directly by binding pathogen-associated molecules. The underlying mechanism remains unknown (Schulenburg and Ewbank, 2007). The results of studies on *S. marcescens* avoidance implicate G protein-coupled chemoreceptors; other candidate pathogen recognition receptors include proteins with leucine-rich repeat domains (Schulenburg et al., 2004) or the large family (>500 members) of F-box domain proteins (Thomas, 2006). Very recently, the role of DCAR1 (dihydrocaffeic acid receptor 1), a G-coupled receptor, has been confirmed by Ewbank's group (Zugasti et al., 2014) in antimycotic response using a genome-wide RNA screen. Although the surface of the nematode *C. elegans* is poorly understood, it is critical for interactions with its surroundings and with pathogens. Recently, Hodgkin's group identified six genes (bus-2, bus-4, bus-12, srf-3, bus-8, and bus-17) encoding proteins predicted to act in surface glycosylation, thereby influencing disease susceptibility (Gravato-Nobre et al., 2011). Mutations in these genes induce resistance to *Microbacterium nematophilum* and perturb the adhesion and biofilm formation of *Yersinia* species, highlighting the importance of interactions with complex surface carbohydrates during infection and biofilm formation processes. A clear, recent example of the potential benefits of this type of study can be found in the work of Alper's group (De Arras et al., 2013). Focusing on host-pathogen interactions, they performed comparative RNA-interference screens in the nematode *C. elegans* and in mouse macrophages. Specifically, they analyzed molecular candidates necessary to recognize pathogens through the LPS (lipopolysaccharide) ligand. Using RNAi, they showed that nearly every gene in this network modulates the response to LPS in mouse macrophage cell lines.

In *C. elegans*, only one Toll homolog (TOL-1) has been identified until now. In 2008, Tenor and Aballay (Tenor and Aballay, 2008) showed that TOL-1 is necessary to avoid *Salmonella enterica* invasion through the pharynx, a first line of defense against pathogens in *C. elegans*. They also demonstrated that TOL-1 is required for the expression of a defensin-like molecule (ABF-2) and a heat-shock protein (HSP-16.41) that is a member of the HSP family proteins needed for *C. elegans* immunity. Thus, for the first time, TOL-1 has been shown to play a direct role in the defense of *C. elegans* against pathogens.

One notable pathway activated by the cell host during pathogen invasion is the mitogen-activated protein kinase signaling pathway. The MAPK signaling mediated innate immunity in *C. elegans* during *S. enterica* infection (Aballay et al., 2003). The *C. elegans* homolog of P38 mitogen-activated protein kinase (MAPK), which is encoded by the pmk-1 gene, is a prerequisite for activation of the Salmonella-induced programmed cell death (PCD). Inactivation of pmk-1 using RNAi completely attenuated Salmonella-elicited PCD. The same group confirmed the importance of the P38 MAP kinase pathway in the *C. elegans* immune response (Pukkila-Worley et al., 2012).

One well-described mechanism that regulates aging in *C. elegans* is the DAF-2 mediated pathway; “the abnormal Dauer formation/insulin-like growth factor (DAF-2/IGF) pathway.” DAF-2 activity shortens life span through its inhibition of DAF-16, a forkhead transcription factor. Using microarrays, Kenyon's group (Murphy et al., 2003) has shown that several DAF-16 targets are antimicrobial genes, as well as genes encoding saposins (related to NK-lysin) and thaumatins which also exhibit antimicrobial activity. Other DAF-16 targets are involved in detoxification and resistance to oxidative stress (e.g., glutathione-S-transferase, catalase and superoxide dismutase) or more general anti-stress mechanisms (Ookuma et al., 2003). Daf-2 mutant worms are resistant to infection, particularly by Gram-positive bacteria.

Intrinsic agents, such as antimicrobial peptides, are important to protect the worm against infection, and most of these peptides belong to the Signal Peptide Peptidase (SPP) protein family. In the intestine of *C. elegans*, SPP-5 exhibits a pore-forming effect on the bacterial membrane and thus kills the bacteria. This antimicrobial polypeptide is needed to deal with *Escherichia coli*, the food source of *C. elegans* in the laboratory, as worms lacking these molecular tools develop poorly due to the substantial number of bacteria that spread throughout their intestines. Certain genes, such as SPP-3, require a contact with particular bacteria to be expressed, whereas others, such as SPP-6, are expressed regardless of the bacteria they get along (Roeder et al., 2010).

*C. elegans* was found to produce reactive oxygen species (ROS) as a powerful defense against infection. Through a combination of studies employing RNA interference and mutants to examine this ROS production (Hoeven et al., 2011), Hoeven and colleagues proposed a theoretical model in which the ROS produced by Ce-Duox1/BLI-3 during infection form part of a protective immune response in the nematode, indicating that ROS production is a conserved, ancient defense mechanism. It is clear from the cited studies that the knowledge on *C. elegans* immune response greatly benefited from RNAi screening approach. The roles of some well-known key factors such as JNK-MAP kinases and DAF-2 protein have been better understood in the immunity context and the remarkable study of Melo and Ruvkun (Melo and Ruvkun, 2012) clearly filled the existing gap between cellular events and behavioral response, showing how molecular pathways coordinate aversion mechanism allowing animals to detect invading pathogens.

### Danio rerio

Morpholino is the most efficient method for gene silencing in *D. rerio* (zebrafish). In zebrafish, four PGRPs have been identified, three of which have been cloned and named *pglyrp-2 pglyrp-5, (pgrp-sc)*, and *pglyrp-6* (Li et al., 2007); these genes encode 6 PGRPs (Chang et al., 2007). Zebrafish PGRPs share common features with mammals PGRPs (Table 1); they possess both amidase and broad-spectrum bactericidal activities. *In vitro*, zebrafish PGRPs exhibit bactericidal activity against both Gram-negative and Gram-positive bacteria. *In vivo*, pglyrp-5 has been identified using morpholino knockdown to be an essential component of the host defense against *Salmonella typhimurium* and *Bacillus subtilis* in the absence of adaptive immunity in the zebrafish embryo. The intracellular signaling pathway downstream of this receptor has been described, indicating that *pglyrp-5 (pgrp-sc)* is not only linked to the immune response but also to apoptosis and developmental processes (Chang et al., 2009).

The adaptor protein MyD88 plays a role in signal transduction downstream from the recognition of pathogens by TLR. In zebrafish, MyD88 morphants are more susceptible to a strain of avirulent *Salmonella typhimurium* (van der Sar et al., 2006). Similarly to mammals, the zebrafish MyD88 signaling pathway causes induction of *il1b* and interferon (*ifnphi1*) (Stockhammer et al., 2009). In terms of TLRs homologous to mammalian cell surface and endosomal TLRs, zebrafish has specific TLRs (*Tlr19, Tlr20a/b, Tlr20f, Tlr21, Tlr22*) that are present on the endosome (Matsuo et al., 2008; Keestra et al., 2010; Meijer and Spaink, 2011). Traf mediates signal transduction from members of the TNF receptor superfamily.

Unlike mammals, zebrafish possess two type II (γ) IFNs: Ifn-γ1 (*ifng1-1*) and Ifn-γ 2 (*ifng1-2*). Morpholino knockdown studies have shown a partially redundant function of *ifng1-1* and *ifng1-2* in mediating resistance of the zebrafish embryo to *Escherichia coli* and *Yersinia ruckeri* infections (Sieger et al., 2009). IFN-γ has been demonstrated to be the major inducer of ROS production in mice and humans. Furthermore, the role of ROS in pathogen killing was first suggested by the observation that patients with chronic granulomatous disease, who have increased susceptibility to infections, were found to produce little or no superoxide radicals (Curnutte and Babior, 1974). In the zebrafish, a tissue-scale gradient of H_2_O_2_ is formed following wound induction (Niethammer et al., 2009). Using morpholino knockdown, researchers have shown that H_2_O_2_ is necessary for the migration of leukocytes to the wound site, particularly due to its known antiseptic role. In this context, the function of ROS production has been shown in zebrafish larvae from knockdown of the NADPH oxidase family member dual oxidase (*duox*) (Flores et al., 2010); *duox* morphants are unable to control enteric *Salmonella typhimurium* infection. In 2010, Phennicie and colleagues reported that cystic fibrosis transmembrane conductance regulator (*cftr*) knockdown disturbs ROS production, which increases the bacterial burden during *Pseudomonas aeruginosa* infection of zebrafish embryos (Phennicie et al., 2010). The role of TNF receptor signaling in resistance to *Mycobacterium marinum* has been described using knockdown in a zebrafish embryo model (Clay et al., 2008). Disruption of TNF receptor expression increases bacterial growth and accelerates granuloma formation. Because granuloma has been shown to play a critical role in tissue dissemination of *Mycobacterium marinum* (Clay et al., 2007), it appears clear that TNF signaling is protective during early stages of infection in the absence of adaptive immunity.

### Planarian

Over the last several years, planarians have become a favored model system for studying regeneration and development (Elliott and Sánchez Alvarado, 2012). Studies of these organisms have provided invaluable insights into the mechanisms of tissue growth and regeneration. Twenty-two years ago, a pioneering study suggested that planarians exhibit a phagocytic response to heat-killed bacteria (Morita, 1991). However, no further efforts were made to carry out a more in-depth molecular characterization of planarian immunity.

Quite recently, Abnave et al. (2014) examined and documented the extraordinary capacity of planarians to destroy a wide range of pathogenic bacteria. Using an RNAi screening technique on *Dugesia Japonica* species, they identified 18 antibacterial resistance factors and highlighted the gene *MORN2*, which is conserved in *Homo sapiens* but has been lost in the *C. elegans* and *D. melanogaster*. Functional analysis of the *MORN2* gene in macrophages demonstrated the role of human MORN2 (Hs-MORN2) in restricting bacterial intracellular growth through non-canonical phagocytosis. This work established planarians as a suitable model organism for identifying anti-bacterial immune factors. Out of 18 antibacterial resistance genes identified they found the human orthologs for half of the genes including MORN2. In planarians, MORN2 protected against all strains of bacteria tested. Combining genetic and functional screen in planarians, researchers ascribed the function of human MORN2 to phagocytosis-mediated restriction of *S. aureus, L. pneumophila* and *M. tuberculosis* growth in macrophages. Further analyses supported the importance of MORN2 expression in promoting LC3-associated phagocytosis of *M. tuberculosis* and the targeting of bacteria to cathepsin-D-positive phagolysosomes. Complementary to these findings, MORN2 co-immunoprecipitated with LC3 and promoted the lipidation of LC3-I in cells challenged by autophagy-inducing stressors: LPS, IFN-γ and starvation.

## Conclusion

Genetic screening in model organisms have proved to be a powerful and valuable tool in researcher's hand, and several remarkable studies on model organism (Tobin et al., 2010, 2012) have helped to understand fundamental principles of vertebrates resistance to infection. Few methods have been as important in changing biology research as the RNAi technique, and it can truly be considered one of the most important technological breakthroughs in modern biology. Today, RNAi methods have greatly evolved, and high-throughput screening can be used to identify and functionally assess the thousands of genes within a genome that potentially participate and tune biological processes. Moreover, as the molecular machinery required for this RNAi technique is naturally present in the cells and is also highly conserved throughout different species, we can readily apply this technique on various animal models.

Study of the immune response process has greatly benefited from the application of RNAi screens, as this approach is most successful when it incorporates a focused search for predicted pathway-regulatory proteins; moreover, the ability to adapt the RNAi method to several species, broadening the spectrum of meaningful data, has allowed researchers to deepen knowledge of the immune system in a way that was never possible before.

### Conflict of interest statement

The Guest Associate Editor, Philippe Soubeyran, declares that, despite having collaborated with author, Eric Ghigo, the review process was handled objectively. The authors declare that the research was conducted in the absence of any commercial or financial relationships that could be construed as a potential conflict of interest.
